# Hypoestoxide reduces neuroinflammation and α-synuclein accumulation in a mouse model of Parkinson’s disease

**DOI:** 10.1186/s12974-015-0455-9

**Published:** 2015-12-18

**Authors:** Changyoun Kim, Emmanuel Ojo-Amaize, Brian Spencer, Edward Rockenstein, Michael Mante, Paula Desplats, Wolf Wrasidlo, Anthony Adame, Emeka Nchekwube, Olusola Oyemade, Joseph Okogun, Michael Chan, Howard Cottam, Eliezer Masliah

**Affiliations:** Department of Neuroscience, University of California, San Diego, La Jolla, CA 92093 USA; Pathology, School of Medicine, University of California, San Diego, La Jolla, CA 92093 USA; Moores Cancer Center, University of California, San Diego, 2880 Torrey Pines Scenic Drive, La Jolla, CA 92037 USA; Immune Modulation, Inc., P.O. Box 998, Bloomington, CA 92316-0998 USA

**Keywords:** Hypoestoxide, Parkinson’s disease, Neuroinflammation, Neurodegeneration, α-synuclein, NF-κB

## Abstract

**Background:**

Deposition of α-synuclein and neuroinflammation are key pathological features of Parkinson’s disease (PD). There is no cure for the disease; however, targeting the pathological features might be available to modulate the disease onset and progression. Hypoestoxide (HE) has been demonstrated as a NF-κB modulator, thereby acting as a potential anti-inflammatory and anti-cancer drug.

**Methods:**

In order to assess the effect of HE in a mouse model of PD, mThy1-α-syn transgenic mice received intraperitoneal (IP) injections of either vehicle or HE (5 mg/kg) daily for 4 weeks.

**Results:**

Treatment of HE decreased microgliosis, astrogliosis, and pro-inflammatory cytokine gene expression in α-syn transgenic mice. HE administration also prevented the loss of dopaminergic neurons and ameliorated motor behavioral deficits in the α-syn transgenic mice, and α-synuclein pathology was significantly reduced by treatment of HE. In addition, increased levels of nuclear phosphorylated NF-κB in the frontal cortex of α-syn transgenic mice were significantly reduced by HE administration.

**Conclusions:**

These results support the therapeutic potential of HE for PD and other α-synuclein-related diseases.

**Electronic supplementary material:**

The online version of this article (doi:10.1186/s12974-015-0455-9) contains supplementary material, which is available to authorized users.

## Background

Intraneuronal Lewy bodies (LBs) and Lewy neurites (LNs) are key pathological features of Parkinson’s disease (PD) [1]. α-synuclein is a small neuronal protein (140 amino acids), and the pathological amyloid fibrils are a major component of LBs and LNs [2]. While the physiological functions of α-synuclein are still unclear, studies have suggested its roles in neuroplasticity and synaptic vesicle recycling [2]. Furthermore, accumulating evidence has demonstrated that abnormal deposition of α-synuclein is not only a pathological feature but also plays critical roles in the onset and progression of diseases [3].

In addition to α-synuclein deposits, neuroinflammation is another pathological feature of PD [4]. For example, accumulations of reactive microglia have been found in the brains of PD patients, and elevated levels of inflammatory cytokines, such as TNFα and IL6 have been detected in the CSF and plasma of PD patients [5]. In addition, studies have shown that neuroinflammation is not only a pathological feature but also plays critical roles in neurodegeneration. Administration of anti-inflammatory drugs prevented dopamine neuronal degeneration in substantia nigra (SN) of toxicant-induced animal models of PD [6]. Epidemiological studies have suggested that administration of non-steroidal anti-inflammatory drugs reduce the risk for PD [7–9] suggesting that reducing or preventing inflammation may reduce the risk of PD.

Microglia, a brain resident immune cell, plays a central role in the process of neuroinflammation. Microglia could be activated by various types of stimuli resulting in neuroinflammation, including systemic inflammation, brain injury, and ischemia [10]. Recent studies have also showed that extracellular α-synuclein can also induce activation of microglia. Exposure to various forms of recombinant α-synuclein can induce activation of microglia [3]. In our previous study, we demonstrated that neuron-released oligomeric forms of α-synuclein-induced microglia activation via interaction with TLR2 and β1-integrin on the surface of microglia [8, 11].

Although it is well established that the outcome of chronic neuroinflammation is neurodegeneration [6], recent studies have also suggested the roles of neuroinflammation in the deposition and accumulation of amyloid protein aggregates in the brain. For example, neuroinflammation is induced by systemic administration of lipopolysaccharide (LPS)-induced accumulation of amyloid-β in hippocampus of non-transgenic (non-tg) mice [12, 13]. Similarly, intraperitoneal injection of LPS increased accumulation of α-synuclein aggregates in substantia nigra of both non-tg and α-synuclein transgenic (α-syn-tg) mice [14]. Furthermore, inhibition of neuroinflammatory enzymes, such as NADPH oxidase and iNOS decreased nigral α-synuclein deposition in α-syn-tg mice [15] once again suggesting that blocking or preventing inflammation may prevent PD pathology.

Hypoestoxide (HE) is a natural diterpene isolated from the shrub *Hypoestes rosea* (Acanthaceae) [16]. Studies have demonstrated that HE may modulate the activity of NF-κB through IκB kinase inhibition [16]. Thereby, HE has been suggested as a potential anti-inflammatory and anti-cancer drug [16, 17]. The polar surface area for HE is 68.4 Å^2^ which is considered very good for blood brain barrier penetration. Therefore, we examined the potency of HE as an anti-neuroinflammatory drug for PD using a mouse model. In conclusion, administration of HE ameliorates neuroinflammation, neurodegeneration, and behavioral defects in a PD mouse model via modulation of NF-κB activity, thus supporting a role for HE as an anti-inflammatory drug for the treatment of PD.

## Methods

### Antibodies and chemicals

The protease and phosphatase inhibitor cocktails were purchased from Sigma-Aldrich (St Louis, MO). Hypoestoxide was obtained from Immune Modulation, Inc. (Bloomington, CA). The following antibodies were used: α-synuclein (Syn-1; BD Bioscience, San Diego, CA); TNFα, glial fibrillary acidic protein (GFAP) (GA5), TH, and NeuN (Millipore, County Cork, Ireland); β-actin (Sigma-Aldrich, St Louis, MO); NF-κB p65 and phospho-NF-κB p65 (Cell signaling, Beverly, MA); IL-1β and IL6 (Abcam, Cambridge, MA); α-synuclein (CT, Syn105) [18]; α-synuclein (syn211) (Life Technologies, Grand Island, NY); and Iba-1 (Wako, Richimond, VA).

### Animal treatment and behavioral analysis

Mice overexpressing human α-synuclein under mThy1 promoter (α-syn-tg) were used for this study because mice develop behavioral motor deficits, axonal pathology, and accumulation of α-synuclein in cortical and subcortical regions, thus mimicking PD [19–21]. The procedure for intraperitoneal injection has been described elsewhere [22]. Briefly, 5-month-old non-tg and α-syn-tg female mice were injected intraperitoneally (IP) with either vehicle (40 % captisol) or hypoestoxide (5 mg/kg) daily for 4 weeks. The right hemibrains were post-fixed in phosphate-buffered 4 % PFA at 4 °C for neuropathological analysis, while the left hemibrains were snap-frozen and stored at −70 °C for subsequent protein and messenger NA (mRNA) analysis. All animal procedures were approved by the UCSD Institutional Animal Care and Use Committee.

Following treatment, animals were assessed for gait and coordination using the open field and the round beam tests. As previously described [23], total activity was calculated as total beam breaks in 10 min. The impairment of gait and balance was accessed by round beam analysis. Three consecutive trials of 1 min each were run in 1 day. The numbers of foot slippages and distance traveled were recorded. The total errors on the beam were calculated as foot slips/distance traveled.

### Immunohistochemistry and immunofluorescence and neuropathological analysis

The procedures for immunohistochemical, immunofluorescence, and neuropathological analysis have been described elsewhere [22, 24]. Briefly, blind-coded sagittal brain sections were incubated with primary antibodies at 4 °C for overnight. The next day, sections were incubated with either biotinylated- or FITC-conjugated secondary antibodies and detected with avidin D-HRP HRP (ABC elite, Vector Laboratories, Burlingame, CA) and with Tyramide Signal Amplification Direct system (PerkinElmer, Waltham, MA), respectively.

To determine the neuroinflammation, neurodegeneration, accumulation of α-synuclein, and NF-κB activation, we stained brain sections with Iba-1, GFAP, TNFα, IL-1β, IL6, human α-synuclein, NF-κB, and phosphorylated NF-κB antibodies, respectively. Sections were imaged by Olympus BX41 microscope. All immunoreactivity levels were determined by optical density analysis using Image Quant 1.43 program (NIH) except the immunoreactivity of Iba-1. The cell numbers of Iba-1-positive cells were determined per field (230 μm × 184 μm) of each animal based on cell body recognition using Image Quant 1.43 program (NIH).

### Preparation of tissue extract and Western blot analysis

The procedures for tissue extract preparation and Western blot analysis have been described elsewhere [25]. Briefly, brain homogenates were prepared in the lysis buffer to separate sodium dodecyl sulfate (SDS)-soluble and SDS-insoluble fractions. Chemiluminescence detection and analysis were performed using Versadoc XL imaging apparatus and Quantity One (Bio-rad, Hercules, CA).

### Quantitative polymerase chain reaction

The procedure for quantitative polymerase chain reaction (qPCR) has been described elsewhere [23]. Briefly, total mRNA was extracted from the mice frontal cortex using RNeasy Lipid mini kit (Qiagen, Germantown, MD) and reverse transcribed using SuperScript VILO cDNA synthesis kit (Life Technologies), respectively. Quantitative real-time PCR was performed using TaqMan® Fast Advanced Master Mix (Life Technologies) according to manufacturer’s instruction with gene-specific primers obtained from Life Technologies, such as TNFα (Mm00443258_m1), IL6 (Mm00446190_m1), IL-1β (Mm00434228_m1), and β-actin (Mm00607939_s1). Amplification of DNA products was measured by the StepOnePlus real-time PCR system (Applied Biosystems, Carlsbad, CA). Relative mRNA levels were calculated according to the 2-exp (ΔΔCt) method. All ΔCT values were normalized to β-actin.

### Statistical analysis

GraphPad Prism (GraphPad Software, San Diego, CA) was used for all statistical analysis. All data are presented as means ± s.e.m. All data were analyzed for statistical significance by using either unpaired *t* test or two-way ANOVA (general linear model) followed by Bonferroni’s multiple comparison post-test.

## Results

### Administration of HE decreases neuroinflammation in a mouse model of PD

**Fig. 1:**
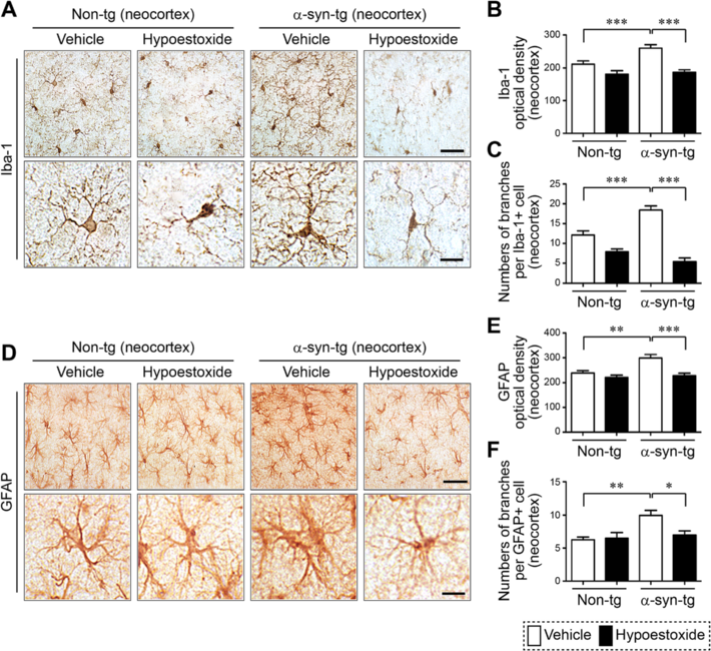
Hypoestoxide reduces neuroinflammation in a mouse model of PD. Immunohistochemical analysis of Iba-1 and GFAP in neocortex of non-tg and α-syn-tg mice injected with either vehicle or hypoestoxide. **a** Immunoreactivity against Iba-1 was analyzed in neocortex of the brains. **b**, **c** The numbers of Iba-1-positive cells (**b**) and the average numbers of branches per Iba-1-positive cell (**c**) (*n* = 5 per each group; two-way ANOVA, Bonferroni’s multiple comparison post-test; ***p < 0.001). *Error bars* represent ±SEM. **d** Immunoreactivity against GFAP was analyzed in neocortex of the brains. **e**, **f** Optical density analysis of GFAP immunoreactivity (**e**) and the average numbers of branches per GFAP-positive cell (**f**) (*n* = 5 per each group; two-way ANOVA, Bonferroni’s multiple comparison post-test; *p < 0.05, **p < 0.01, ***p < 0.001). *Error bars* represent ±SEM. *Scale bars* = 100 μm (low magnification) and 20 μm (high magnification)

Neuroinflammation is a key pathological feature in PD that is recapitulated in the mThy-1α-synuclein transgenic (α-syn-tg) mouse model of PD [18, 23]. In order to determine if neuroinflammation could be ameliorated, we administered HE (5 mg/kg) through intraperitoneal injection (IP) to the α-syn-tg mice daily for 4 weeks. Analysis of the numbers of brain immune cells, such as Iba-1-positive microglia and GFAP-positive astrocytes were significantly increased in the neocortex of α-syn-tg mice (Fig. 1). Interestingly, administration of HE significantly decreased the numbers of microglial cells and the levels of astrogliosis in the neocortex in α-syn-tg mice to levels similar to those in non-tg mice (Fig. 1a, b, d, and e). In addition to a decrease in overall numbers of immune cells in the α-syn-tg mice, we observed a decrease in the numbers of branches per glial cell (Fig. 1a, c, d, and f) [26]. A positive interactive effect of HE treatment on Iba-1 optical density (*F*_interaction (1, 16)_ = 80.48, *p* < 0.0001), numbers of microglia branches (*F*_interaction (1, 16)_ = 83.25, *p* < 0.0001), GFAP optical density (*F*_interaction (1, 16)_ = 15.88, *p* = 0.0011), and numbers of astroglial branches (*F*_interaction (1, 16)_ = 4.04, *p* = 0.0616) was confirmed by two-way ANOVA.

**Fig. 2:**
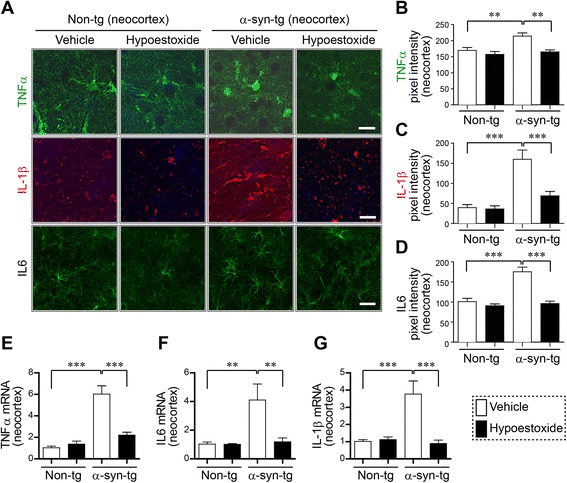
Hypoestoxide reduces pro-inflammatory cytokines in the brain of α-syn-tg mice. **a**–**d** Immunofluorescence or immunohistochemical analysis of TNFα, IL-1β, and IL6 in the neocortex of non-tg and α-syn-tg mice treated with either vehicle or hypoestoxide. **a** Representative images. **b**–**d** Quantitative analysis of fluorescence intensities of TNFα (**b**), IL-1β (**c**), and IL6 (**d**) stainings were analyzed in the neocortex of the brains (*n* = 5 per each group; two-way ANOVA, Bonferroni’s multiple comparison post-test; **p < 0.01, ***p < 0.001). *Error bars* represent ±SEM. **e**–**g** Quantitative real-time PCR analysis of expression levels of TNFα (**e**), IL6 (**f**), and IL-1β (**g**) in the neocortex of mice presented as levels over β-actin (*n* = 4 per each group; two-way ANOVA, Bonferroni’s multiple comparison post-test; **p < 0.01, ***p < 0.001). *Error bars* represent ±SEM. *Scale bar* = 20 μm

To verify our observation, we analyzed the levels of pro-inflammatory cytokines using immunohistochemical analysis and gene expression analysis (Fig. 2). The levels of TNFα, IL-1ß, and IL6 were increased in α-syn-tg mice compared to non-tg mice (Fig. 2a–d). In contrast, treatment of HE significantly reduced the levels of these pro-inflammatory cytokines in the neocortex of α-syn-tg mice (Fig. 2a–d). A positive interactive effect of HE treatment on the levels of TNFα (*F*_interaction (1, 16)_ = 12.34, *p* = 0.0029), IL-1β (*F*_interaction (1, 16)_ = 11.58, *p* = 0.0036), and IL6 (F_interaction (1, 16)_ = 31.06, *p* < 0.0001) was confirmed by two-way ANOVA. In addition, quantitative gene expression analysis showed the mRNA levels of TNFα, IL-1β, and IL6 were clearly decreased by HE administration in the neocortex of α-syn-tg mice (Fig. 2e–g). A positive interactive effect of HE treatment on the mRNA levels of TNFα (*F*_interaction (1, 16)_ = 15.78, *p* = 0.0019), IL-1β (*F*_interaction (1, 16)_ = 11.65, *p* = 0.0051), and IL6 (*F*_interaction (1, 16)_ = 6.40, *p* = 0.0264) was confirmed by two-way ANOVA. Together, these results suggest that administration of HE inhibits activation of microglia and astrocytes, thereby reducing the production of pro-inflammatory cytokines in a mouse model of PD.

### Amelioration of neurodegeneration and behavioral defect by HE administration in a mouse model of PD

**Fig. 3:**
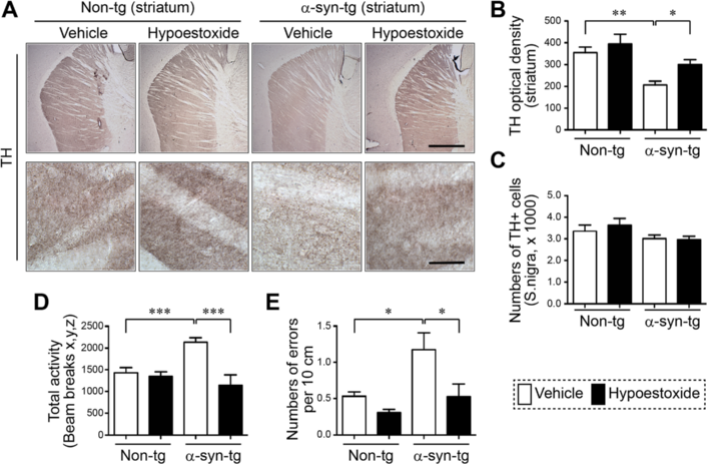
Hypoestoxide ameliorates neurodegeneration and behavioral deficits in α-syn-tg mice. **a**–**c** Immunohistochemical analysis of TH in the striatum and substantia nigra (S.Nigra) of non-tg and α-syn-tg mice treated with either vehicle or hypoestoxide. **a** Representative images of striatum. **b** The levels of dopaminergic fiber in striatum were analyzed by optical density quantification (*n* = 5 per each group; two-way ANOVA, Bonferroni’s multiple comparison post-test; *p < 0.05, **p < 0.01). *Error bars* represent ±SEM. **c** Stereological analysis of TH-positive cells in S. Nigra. **d**, **e** Motor behavioral analysis of non-tg and α-syn-tg mice. Total activity from open field analysis (**d**) and the numbers of slippages from round beam test (**e**) were recorded (*n* = 5 per each group; two-way ANOVA, Bonferroni’s multiple comparison post-test; *p < 0.05, ***p < 0.001). *Error bars* represent ±SEM. *Scale bars* = 250 μm (**a**, upper panel) and 25 μm (**a**, lower panel)

Neuroinflammation is one of the well-known causes of neurodegeneration [3]. Since the levels of pro-inflammatory cytokines were significantly decreased by HE administration in α-syn-tg mice, we hypothesized that administration of HE would prevent neurodegeneration and behavioral deficit through inhibition of neuroinflammation in α-syn-tg mice. To verify the hypothesis, we performed neurodegeneration analysis and behavioral tests using non-tg and α-syn-tg mice treated with either vehicle or HE (Fig. 3). Neuronal overexpression of human α-synuclein resulted in the loss of TH-positive striatal fibers in α-syn-tg mice while the numbers of nigral TH-positive cells were not altered by α-synuclein expression (Fig. 3a–c). However, administration of HE significantly decreased the loss of TH-positive striatal fibers in α-syn-tg mice (Fig. 3a, b). A positive interactive effect of HE treatment on the level of TH-positive striatal fibers (*F*_interaction (1, 16)_ = 5.12, *p* = 0.038) was confirmed by two-way ANOVA.

To investigate the effect of HE on the anxiety-like behavior and motor behavior deficit in α-syn-tg mice, we performed open field and round beam tests, respectively (Fig. 3d, e). α-syn-tg mice showed a significant increase of the beam break numbers and the total round beam errors compared to non-tg control mice. Treatment of α-syn-tg mice with HE reduced these errors to levels observed in non-tg mice (Fig. 3d, e). A positive interactive effect of HE treatment on the beam break numbers (*F*_interaction (1, 16)_ = 15.61, *p* = 0.0011) and total round beam errors (*F*_interaction (1, 16)_ = 8.58, *p* = 0.0098) was confirmed by two-way ANOVA. Taken together, these results suggest that administration of HE prevents neurodegeneration and ameliorates behavioral defect in a mouse model of PD.

### Administration of hypoestoxide results in reduced neuronal α-synuclein accumulation in a mouse model of PD

**Fig. 4:**
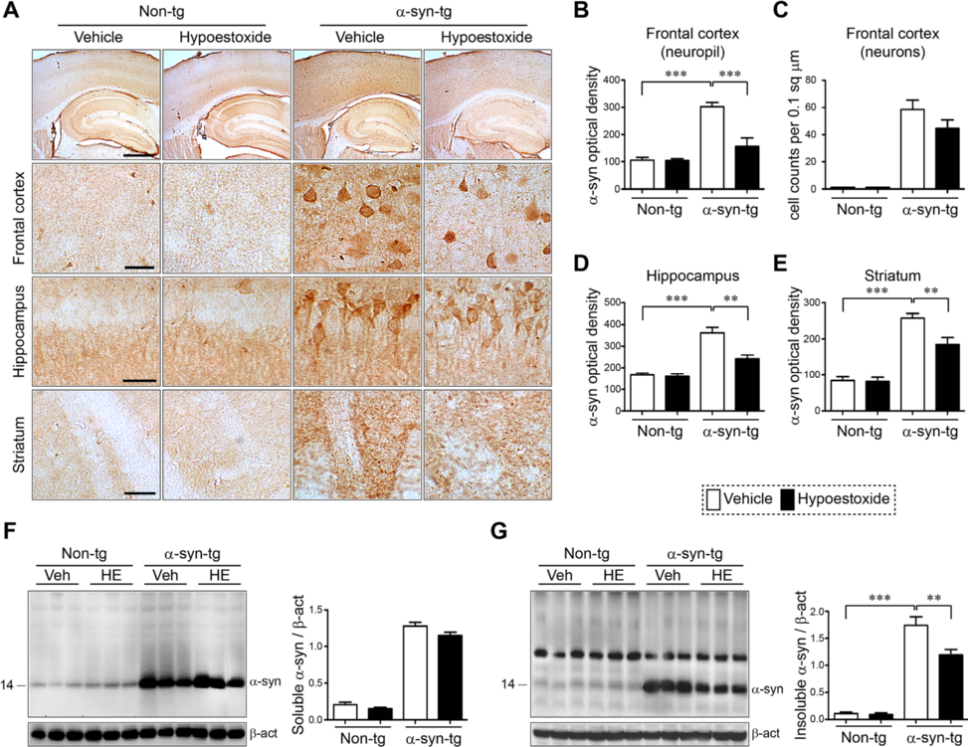
Hypoestoxide reduces deposition of α-synuclein in a mouse model of PD. **a** Representative immunohistochemical staining of α-synuclein in the frontal cortex, hippocampus, and striatum. **b** Optical density analysis for α-synuclein-positive neuropil in the frontal cortex (*n* = 5 per each group; two-way ANOVA, Bonferroni’s multiple comparison post-test; ***p < 0.001). *Error bars* represent ±SEM. **c** The numbers of α-synuclein-positive cells in the frontal cortex. **d**, **e** Optical density analysis of immunoreactivity of α-synuclein in the hippocampus (**d**) and in the striatum (**e**) (*n* = 5 per each group; two-way ANOVA, Bonferroni’s multiple comparison post-test; **p < 0.01, ***p < 0.001). *Error bars* represent ±SEM. **f**, **g** Biochemical analysis of SDS-soluble (**f**) and SDS-insoluble (**g**) fractions from the frontal cortex for α-synuclein (*n* = 5 per each group; two-way ANOVA, Bonferroni’s multiple comparison post-test; **p < 0.01, ***p < 0.001). *Error bars* represent ±SEM. *Scale bars* = 250 μm (low magnification) and 25 μm (high magnification)

To determine if the behavioral improvements observed in the α-syn-tg mice were related to alterations of α-synuclein pathology, we performed immunohistochemical analysis for α-synuclein with brain sections from non-tg and α-syn-tg mice treated with either vehicle or HE. Immunohistochemical analysis showed overexpression of α-synuclein in neurons and the neuropil of α-syn-tg mice (Fig. 4a–e). Surprisingly, administration of HE significantly decreased the levels of α-synuclein in neurons and neuropil in α-syn-tg mice (Fig. 4a–e). A positive interactive effect of HE treatment on the optical density of α-synuclein (frontal cortex, *F*_interaction (1, 16)_ = 30.74, *p* < 0.0001; hippocampus, *F*_interaction (1, 16)_ = 13.66, *p* = 0.0020; striatum, *F*_interaction (1, 16)_ = 7.19, *p* = 0.0164) was confirmed by two-way ANOVA. To confirm our observations, we performed immunofluorescence analysis with human α-synuclein-specific antibodies (Additional file 1). Immunoreactivity against human α-synuclein was not detected in the frontal cortex of non-tg mice, but it was highly detected in the frontal cortex of α-syn-tg mice (Additional file 1a, b). Similar to results from the immunohistochemical analysis, the level of human α-synuclein immunoreactivity was significantly decreased by HE administration in the frontal cortex of α-syn-tg mice (Additional file 1a, b). Recent evidence suggests the C-terminal fragments of α-synuclein are particularly neurotoxic [18]. To determine if the administration of HE affected the accumulation of these C-terminal fragments, we used an antibody that specifically recognizes the C-terminus of human α-synuclein. Immunoreactivity against C-terminus human α-synuclein was also significantly decreased by HE administration in the frontal cortex of α-syn-tg mice (Additional file 1c, d).

To verify our findings, we performed biochemical analysis (Fig. 4f, g). Brain homogenates were separated into SDS-soluble and SDS-insoluble factions, and analyzed by immunoblot analysis. Interestingly, the levels of α-synuclein in SDS-soluble fractions from α-syn-tg mice were not affected by HE administration (Fig. 4f, g). However, the levels of SDS-insoluble α-synuclein were significantly decreased in the brain homogenates from HE-administrated α-syn-tg mice (Fig. 4f, g). A positive interactive effect of HE treatment on the level of SDS-insoluble α-synuclein (*F*_interaction (1, 16)_ = 8.68, *p* = 0.0095) was confirmed by two-way ANOVA. Taken together, these results suggest that administration of HE reduces the accumulation of α-synuclein in a mouse model of PD.

### HE modulates neuroinflammation via inhibition of NF-κB activity in a mouse model of PD

**Fig. 5:**
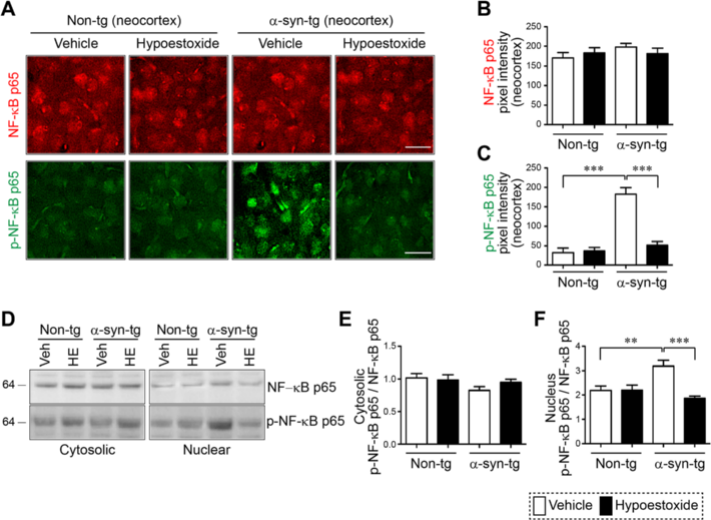
Hypoestoxide inhibits NF-κB signaling in a mouse model of PD. **a** Representative immunohistochemical staining of NF-κB and phosphorylated NF-κB in the neocortex of non-tg and α-syn-tg mice treated with either vehicle or hypoestoxide. **b**, **c** Fluorescence intensities against NF-κB (**b**) and phosphorylated NF-κB (**c**) were analyzed in the neocortex of the brains (*n* = 5 per each group; two-way ANOVA, Bonferroni’s multiple comparison post-test; ***p < 0.001). *Error bars* represent ±SEM. **d** Western blot analysis of cytosolic and nuclear fractions from the neocortex for NF-κB and phosphorylated NF-κB. **e** The relative cytosolic phosphorylated NF-κB levels normalized against cytosolic total NF-κB. **f** The relative nuclear phosphorylated NF-κB levels normalized against total nuclear NF-κB (*n* = 5 per each group; two-way ANOVA, Bonferroni’s multiple comparison post-test; **p < 0.01, ***p < 0.001). *Error bars* represent ±SEM. *Scale bar* = 20 μm

Previous work suggests that HE modulated the activity of NF-κB, a key immune response signaling mediator, through inhibition of IκB kinase in immune cells [16]. Therefore, we investigated the alteration of NF-κB activity in the neocortex of non-tg and α-syn-tg mice that received either vehicle or HE. Immunofluorescence analysis showed that total levels of NF-κB were not changed by HE administration in the neocortex of non-tg or α-syn-tg mice (Fig. 5a, b). However, the level of immunoreactivity against phosphorylated NF-κB, the activated form of NF-κB, was highly elevated (fourfold) in the neocortex of α-syn-tg mice (Fig. 5a, c). In addition, the elevated level of phosphorylated NF-κB was significantly decreased by administration of HE in the neocortex of α-syn-tg mice to levels observed in non-tg mice (Fig. 5a, c). A positive interactive effect of HE treatment on the immunoreactivity of phosphorylated NF-κB (*F*_interaction (1, 16)_ = 27.70, *p* < 0.0001) was confirmed by two-way ANOVA. To verify our findings, we performed biochemical analysis using brain homogenates from the cortex of non-tg and α-syn-tg mice (Fig. 5d–f). Brain homogenates were separated into cytosolic and nuclear fractions by centrifugation, and each fraction was analyzed by Western blot analysis. Total levels of NF-κB were not altered by HE administration in non-tg and α-syn-tg mice (Fig. 5d, e). However, the level of phosphorylated NF-κB was significantly increased only in nuclear fraction from α-syn-tg mice brain homogenates (Fig. 5d, f). Similar to results observed by immunofluorescence, the level of phosphorylated NF-κB was significantly reduced by HE administration in α-syn-tg mice (Fig. 5d, f). A positive interactive effect of HE treatment on the level of phosphorylated NF-κB (*F*_interaction (1, 16)_ = 11.55, *p* = 0.0037) was confirmed by two-way ANOVA. Taken together, these results suggest that administration of HE decreases neuroinflammation through modulation of NF-κB activity in a mouse model of PD.

## Discussion

Previous studies have suggested an anti-inflammatory effect of HE, so we examined the potential for HE as an anti-neuroinflammatory drug in the pathogenesis of PD [16]. The levels of cytokine expression and the number of reactive glial cells were significantly reduced by HE administration in a model of PD. In addition, administration of HE prevented neurodegeneration in a mouse model of PD. The loss of TH-positive neurons was significantly decreased by administration of HE in α-syn-tg mice. Behavioral defect also has been ameliorated by HE administration in α-syn-tg mice. Furthermore, we demonstrated that HE inhibits the activity of NF-κB which results in the decrease of neuroinflammation in α-syn-tg mice.

Neuroinflammation is a typical pathological feature of PD playing a critical role in disease onset and progression [3, 4]. Recent studies have also suggested that extracellular α-synuclein is a strong inducer of neuroinflammation [8, 27]. Neurons endogenously release α-synuclein via unconventional exocytosis, and this secretion could be modulated by multiple factors including genetic defects, oxidative modification of α-synuclein, mitochondrial dysfunction, and autophagy inhibition [28–32]. Previous studies have shown that exposure to various forms of recombinant α-synuclein induces microglia activation [25, 33–35]. In addition, we have demonstrated that oligomeric forms of neuron-released α-synuclein interact with TLR2 and β1-integrin on the surface of microglia, thereby inducing pro-inflammatory responses [8, 11]. Thus, in PD and other synucleinopathies, reducing neuroinflammation may be a target for therapeutic intervention.

Intraneuronal accumulations of α-synuclein aggregates are typical pathological features of PD, and studies have demonstrated that these deposits are not only pathological but also play a critical role in the onset and development of PD. Recent studies have shown that neuronal accumulation of α-synuclein can be affected by multiple intra- and extra-neuronal factors, including genetic defects, dysfunction of protein quality control systems, secondary structural alterations, and exposure to environmental toxicants [2, 24]. In addition, neuroinflammation has been suggested as a promotable factor for α-synuclein aggregates in neurons [3]. In this study, we observed that administration of HE reduced the neuronal accumulation of α-synuclein in a model of PD. Since the levels of α-synuclein mRNA were not affected by administration of HE (data not shown), we speculate that activation of the intraneuronal autophagy process can be regulated by neuroinflammation.

We still do not know the mechanism by which microglia-mediated neuroinflammation affects neuronal accumulation of α-synuclein aggregates. However, recent studies have shown that some pro-inflammatory cytokines inhibit autophagy, an efficient intracellular process for α-synuclein elimination [2, 36]. For example, IL-10 inhibits starvation-induced, rapamycin-induced, and lipopolysaccharide-induced autophagy in murine macrophages [37–39]. In addition, IL-4 and IL-13 inhibit both starvation-induced and IFN-γ-induced autophagosome formations in human and murine macrophages [40]. These observations suggest that cytokines from activated microglia may inhibit the autophagy process of neighboring neurons in the brain, thereby resulting in a neuronal α-synuclein accumulation. Considering the previous results together with our current study, we speculate that administration of HE decreases neuronal accumulation of α-synuclein via reduction of neuroinflammation in a model of PD leading to increased autophagic degradation of α-synuclein.

## Conclusions

In conclusion, our work suggests that administration of HE modulates the activity of NF-κB in a model of PD; therefore, HE may be a potent anti-PD drug that can reduce neuroinflammation, neurodegeneration, and α-synucleinopathy.

## Additional file

Additional file 1:
**Hypoestoxide reduces human α-synuclein accumulation in a mouse model of PD.** Mice brain sections were inmmunostained against human α-synuclein (Syn211 antibody) or C-terminal of human α-synuclein (Syn105 antibody). **a** Immunofluorescence analysis of human α-synuclein in the frontal cortex of non-tg and α-syn-tg mice treated with either vehicle or hypoestoxide. (*n* = 5 per group; unpaired *t* test; **p* < 0.05). Error bars represent ± SEM. **b** Fluorescence intensity against human α-synuclein was analyzed in frontal cortex of the brains. **c** Immunohistochemical analysis of C-terminal of human α-synuclein in the frontal cortex of non-tg and α-syn-tg mice. **d** Optical density analysis for C-terminal of α-synuclein in frontal cortex. (*n* = 5 per group; unpaired *t* test; ***p* < 0.01). Error bars represent ± SEM. Scale bars = 250 μm (low magnification) and 25 μm (high magnification). (TIF 13692 kb)

## Abbreviations

ANOVA: analysis of variance
GFAP: glial fibrillary acidic protein
Iba1: ionized calcium-binding adapter molecule 1
IL: interleukin
TH: tyrosine hydroxylase
TNFα: tumor necrosis factor α
