# Supplementary figures and images for: Hypoestoxide reduces neuroinflammation and α-synuclein accumulation in a mouse model of Parkinson’s disease
Source: J Neuroinflammation. 2015 Dec 18;12:236. doi: 10.1186/s12974-015-0455-9 (PMC4683943; doi:10.1186/s12974-015-0455-9)

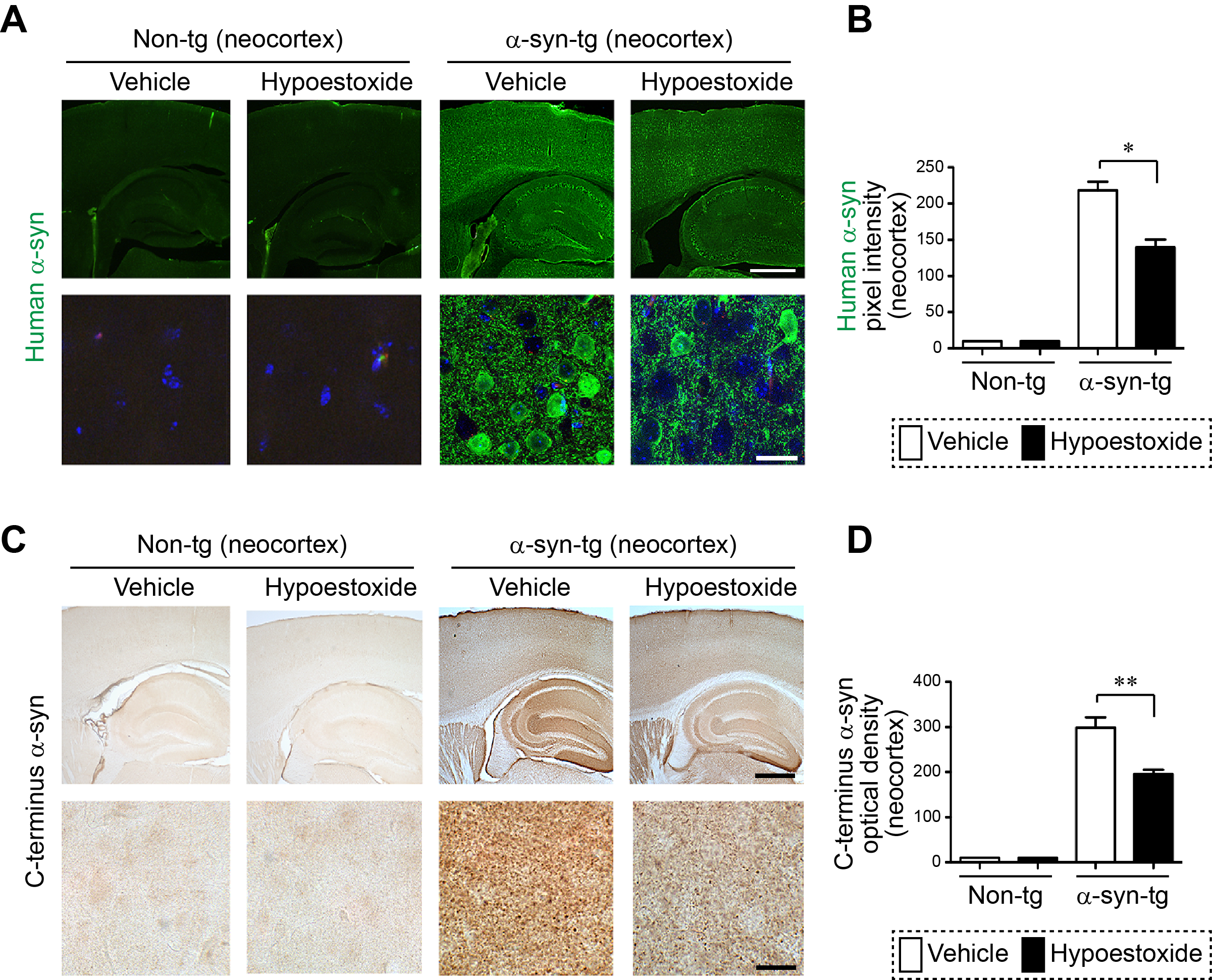

Supplement: Additional file 1: — Hypoestoxide reduces human α-synuclein accumulation in a mouse model of PD. Mice brain sections were inmmunostained against human α-synuclein (Syn211 antibody) or C-terminal of human α-synuclein (Syn105 antibody). a Immunofluorescence analysis of human α-synuclein in the frontal cortex of non-tg and α-syn-tg mice treated with either vehicle or hypoestoxide. (n = 5 per group; unpaired t test; *p < 0.05). Error bars represent ± SEM. b Fluorescence intensity against human α-synuclein was analyzed in frontal cortex of the brains. c Immunohistochemical analysis of C-terminal of human α-synuclein in the frontal cortex of non-tg and α-syn-tg mice. d Optical density analysis for C-terminal of α-synuclein in frontal cortex. (n = 5 per group; unpaired t test; **p < 0.01). Error bars represent ± SEM. Scale bars = 250 μm (low magnification) and 25 μm (high magnification). (TIF 13692 kb) [file 12974_2015_455_MOESM1_ESM.tif]
